# Cadmium Impairs p53 Activity in HepG2 Cells

**DOI:** 10.1155/2014/976428

**Published:** 2014-03-13

**Authors:** C. Urani, P. Melchioretto, M. Fabbri, G. Bowe, E. Maserati, L. Gribaldo

**Affiliations:** ^1^Department of Earth and Environmental Sciences, University of Milano Bicocca, piazza della Scienza 1, 20126 Milan, Italy; ^2^Institute for Health and Consumer Protection, Joint Research Centre, Via Enrico Fermi 2749, 21027 Ispra, Italy; ^3^Dipartimento di Medicina Clinica e Sperimentale, Università dell'Insubria, 21100 Varese, Italy

## Abstract

Cadmium and cadmium compounds are contaminants of the environment, food, and drinking water and are important constituents of cigarette smoke. Cd exposure has also been associated with airborne particulate CdO and with Cd-containing quantum dots in medical therapy. Adverse cadmium effects reported in the literature have stimulated during recent years an ongoing discussion to better elucidate cadmium outcomes at cell and molecular level. The present work is designed to gain an insight into the mechanism of p53 impairment at gene and protein level to understand Cd-induced resistance to apoptosis. We used a hepatoma cell line (HepG2) derived from liver, known to be metal responsive. At genotoxic cadmium concentrations no cell cycle arrest was observed. The p53 at gene and protein level was not regulated. Fluorescence images showed that p53 was correctly translocated into the nucleus but that the p21^Cip1/WAF-1^, a downstream protein of p53 network involved in cell cycle regulation, was not activated at the highest cadmium concentrations used. The miRNAs analysis revealed an upregulation of mir-372, an miRNA able to affect p21^Cip1/WAF-1^ expression and promote cell cycle progression and proliferation. The role of metallothioneins and possible conformational changes of p53 are discussed.

## 1. Introduction 

Cadmium (Cd) is a toxic element present in air, soil, sediment, and water. It is released into the environment through the waste from heavy metal mining, manufactures of nickel-cadmium batteries, and from other industrial and agricultural activities. It is ubiquitously present in the environment and in food, thus leading to a potential risk of human exposure. Nonoccupational exposure is mainly from diet and smoking, due to an accumulation of Cd in tobacco plants [1]. More recently, Cd exposure has been associated with airborne particulate CdO and with Cd-containing quantum dots in medical therapy [2, 3]. Targets of Cd toxicity include liver, lung, kidney cortex, bone, the cardiovascular system, and the immune system ([4], see the reviews [5–7]).

Cd and Cd compounds have been classified as human carcinogens (Group 1) by the World Health Organization's International Agency for Research on Cancer [8] and by the National Toxicology Program [9]. Although Cd carcinogenicity has been recognized by epidemiological studies and animal experiments, the underlying mechanisms are still matter of research activities. Proposed mechanisms have been recently reviewed [10, 11] and range from thiol-containing protein affection and consequent production of reactive oxygen species, and interference with essential metals. Moreover, the deregulation of the cellular response to DNA damage and the resistance to apoptosis are among other proposed mechanisms involved in Cd-induced carcinogenicity [5].

The tumor suppressor protein p53 is a crucial component of the cellular response to DNA damage, and it is primarily involved in defense mechanisms by transcriptional activation of genes responsible of growth arrest and apoptosis for the elimination of heavily damaged cells. The inactivation of p53 is one common feature found in human cancers [12]. Cd has been demonstrated to interfere with the structure and function of p53 [5], although opposite effects have been reported. Namely, some authors refer to the induction of the p53-mediated stress response [13], while others demonstrated an inactivation of p53 via structural changes [14].

In this study we focused on the p53 pathway at gene and protein level to better investigate the involvement of this tumor suppressor protein in Cd-induced carcinogenesis. A microRNA analysis was further performed to evidence a possible role of these small and noncoding regulatory molecules.

We used the HepG2 cells as a model of human origin from a target organ. The HepG2 cells are able to form structures typical of normal hepatocytes, express liver-specific functions, are metal responsive, and, more in general, are well characterized, thus making them a useful model for mechanistic studies on Cd carcinogenicity [15–19].

## 2. Materials and Methods

### 2.1. Cell Line and Culture Conditions

HepG2 cells were routinely grown in a monolayer culture in the presence of Opti-MEM medium (Invitrogen, San Giuliano Milanese, MI, Italy) supplemented with 10% heat inactivated fetal bovine serum (Invitrogen) and 1% antibiotics. The cells were maintained in an incubator at 37°C and humidified atmosphere of 5% CO_2_. The medium was replaced twice a week and the cells were trypsinized and diluted every 7 days at 1 : 3 ratio. The cells were transferred either into 165 cm^2^ flasks (Costar, Euroclone, Pero, MI, Italy) for protein preparations and cell cycle analysis, or into 8 cm^2^ plastic dishes (Costar) for immunofluorescence analysis. Cells grown in complete culture medium represented the controls. A 1 mM stock solution of CdCl_2_ monohydrate 97% purity (Cd, BDH Italia, Rome, Italy) was prepared in sterile MilliQ water (Millipore, Vimodrone, MI, Italy), and further dilutions were prepared in complete culture medium just before use.

### 2.2. Cell Cycle Analysis

The cells were seeded (7 × 10^5^ cells/Petri dishes) and treated (10 *μ*M Cd, 24, 48, and 72 hours) 24 h after seeding. Treated and control cells were harvested by trypsinization, collected by centrifugation (200 g, 5 min), and washed in PBS. The samples were further centrifuged (200 g, 5 min) to remove the PBS, fixed in cold ethanol, and stored at −20°C until use. Defrosted samples were centrifuged (200 g, 10 min) to remove ethanol and were incubated (15 min) for RNA hydrolysis and DNA staining using the DNA Prestain kit (Coulter Reagents, Beckman Coulter). Cell cycle distribution was analyzed by flow cytometry (Becton Dickinson Italia, Buccinasco, MI, Italy). The percentage of cells in each phase was estimated by FlowJo software using the Dean-Jett-Fox best fit and compared to untreated control cells.

### 2.3. p53 Extraction and Immunochemical Analysis

HepG2 cells (5 × 10^6^ cells/flask) were exposed to increasing Cd concentrations (0.1–10 *μ*M) for 24 h. At the end of the exposure time, the cells were harvested by trypsinization, collected by centrifugation (200 g, 10 min), and washed with PBS to remove Cd excess. The cells were collected again by centrifugation (200 g, 10 min), and homogenates of total proteins were obtained by resuspension in sample buffer (0.05 M Tris-HCl, pH 6.8, containing 2% SDS, 10% glycerol, 10% *β*-mercaptoethanol) containing 1 mM PMSF freshly added prior use. Homogenates were boiled for 5 min, passed 3-4 times through a syringe needle (22 ga *Ø*), and stored at −80°C until use. Total protein content was estimated by the Lowry method [20] using BSA as a standard.

Fifty *μ*g of total proteins was separated on 10% NuPAGE gels (Invitrogen) in MOPS running buffer (Invitrogen, cat. number NP0001) and transferred onto a nitrocellulose membrane and processed for immunochemical analysis. A p53 monoclonal antibody (StressGen, Vinci-Biochem, Vinci, FI, Italy) was used, and the membrane incubation was run overnight at 4°C. After incubation with the secondary antibody (mouse IgG alkaline phosphatase conjugate, Sigma), the specific bands were visualized by the colorimetric substrates BCIP/NBT (Fast, Sigma).

### 2.4. p53 Immunofluorescence Localization

HepG2 cells were plated (100.000 cells/cm^2^) on glass coverslips and left to recover at 37°C in incubator. The next day, the cells were treated with Cd (2, and 10 *μ*M) for 24 h. After treatment, the cells were rinsed with PBS, fixed with methanol (5 min, −20°C), and processed for immunofluorescence labeling of the p53 protein. Primary antibody mouse anti-p53 (SterssGen) at 1 : 50 dilution in PBS + 1% BSA was used, and the cells were incubated for 1 h at 37°C. After saturation with PBS and PBS + 1% BSA, the cells were labeled with 1 : 100 Alexa Fluor 594 (Molecular Probes, Invitrogen) and incubated for 45 min at 37°C in humidified atmosphere. Then, samples were washed in PBS and distilled water, stained with DAPI (4′,6-diamidino-2-phenylindole) for nuclei visualization, and air-dried prior mounting. The coverslips were viewed on a Zeiss Axioplan microscope equipped with epifluorescence optics and a digital camera (CoolSnap-ProColors Media Cybernetics, Bethesda, MA, USA), and images were taken and stored using the Image Proplus software (Media Cybernetics).

### 2.5. p21^Cip1/WAF-1^ Immunochemical Analysis

HepG2 homogenates obtained as described in* par.2.4* were used to evaluate the p21^Cip1/WAF-1^ expression in Cd-treated cells (0.1–10 *μ*M). Fifty *μ*g of homogenated proteins was separated by electrophoresis in 12% NuPage gels (Invitrogen) and processed as previously described [16] to enhance membrane transfer and retention of low molecular weight proteins. Mouse anti-p21^Cip1/WAF-1^ was used as a primary antibody (Invitrogen). Anti-mouse alkaline phosphatase conjugate (Sigma, St. Louis, MO, USA) was used as a secondary antibody, and protein binding was visualized by the colorimetric substrate BCIP/NBT (Sigma-Aldrich, Milano Italy).

### 2.6. MicroRNA Expression Profiling

RNA was extracted using the MIRVANA kit (AMBION) according to the manufacturer's instructions. Concentration and quality were determined by Nanodrop. Total RNA was reverse transcribed with Taqman MicroRNA Reverse Transcription Kit using Megaplex RT Primers (Applied Biosystems). Real-time PCR reactions were carried out on preconfigured microfluidic cards (Taqman Array MicroRNA Cards, set A, V2.2 and set B, V3, Applied Biosystems) allowing the detection of about 754 unique assays specific and four candidate endogenous control assays.

Two biological replicates for control and two for 10 *μ*M Cd were tested. Experimental data were then analyzed by SDS 2.3 software (Applied Biosystems) and the relative expression values were calculated using as endogenous control U6 for miRNA. MiRNAs with a threshold cycle <33 that showed a log fold change greater than one in samples treated with cadmium as compared to control samples were considered as induced.

### 2.7. Statistical Analysis

Student's *t*-test or ANOVA (multiple range test) was used for comparisons. The software package Statgraphics Plus version 5.0 (Statistical Graphics Corp., Manugistic Inc. Rockville, MD, USA) was used for the statistical analysis.

## 3. Results

### 3.1. Cell Cycle Progression Is Not Affected by Cd Exposure in HepG2 Cells

We previously demonstrated by MTT assay that Cd concentrations used in the present work induce a maximum of 20% loss of viability at 10 *μ*M Cd. The IC_50_ value (50% of the reduction of cell viability) was computed to be 25.5 *μ*M [16].

Cadmium induces DNA damage in HepG2 cells, as previously reported [22, 23], and as confirmed by our experiments on single-strand breaks formation (data not shown).

In the presence of DNA damage (e.g., single strand breaks), the cell systems have evolved multiple mechanisms to avoid damage propagation. Among these, responses working in mammalian cells include the cell cycle check points control mechanisms, as well as apoptosis for the elimination of heavily damaged cells.

**Figure 1:**
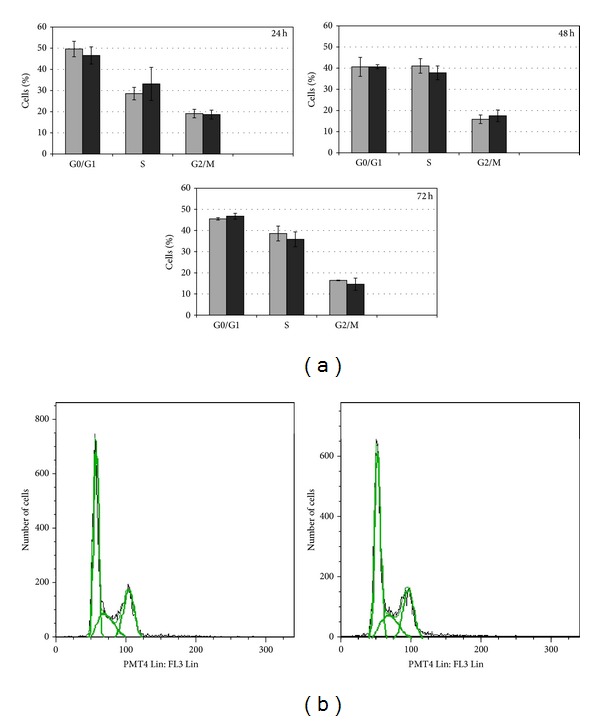
Effects of CdCl_2_ on cell cycle distribution of HepG2 cells. HepG2 cells were cultured in the presence of 10 *μ*M Cd concentration for different time points (24, 48, and 72 h). (a) The distribution of the cell population in the different cell cycle phases of treated cells (black bar) is always comparable to controls (grey bar) at all tested time points. In (b) an example of histogram obtained by flow cytometer analysis is displayed; the DNA content (*x* axis) is plotted against the cell number (*y* axis). Cells from three independent experiments were analyzed.

Our results showed that in HepG2 cells exposed to the highest Cd concentration (10 *μ*M) for different time points (24, 48, and 72 h), there was no effect on cell cycle progression. The distribution of cell population among the cell cycle phases (G1, S, G2/M) did not change when statistically compared to controls (Figure 1(a)), even after 72 h of Cd exposure (Figures 1(a) and 1(b)).

### 3.2. p53 Expression and Localization in Cd-Stressed HepG2 Cells

**Figure 2:**
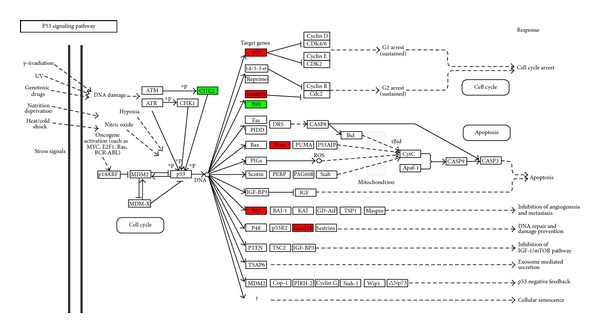
Representation of the p53 signaling pathway map from KEGG. The genes identified as regulated in 10 *μ*m Cd-treated HepG2 cells are colored in the p53 signaling pathway map. The genes in red are upregulated and those in green are downregulated. Genes identified as regulated have a false discovery rate corrected *P* value smaller than 0.05 and fold change greater than 2.

**Figure 3:**
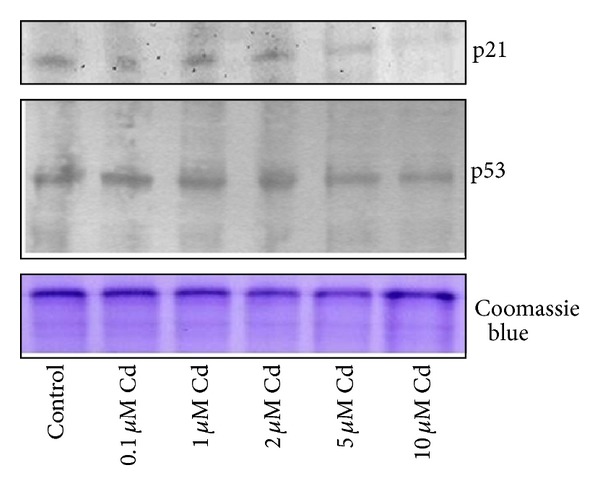
Effect of CdCl_2_ on p53 and p21^Cip1/WAF-1^ protein expression. HepG2 homogenated proteins (50 *μ*g/lane) were separated on 12% gels or 10% gels, transferred onto nitrocellulose membrane, and probed for p21^Cip1/WAF-1^ or p53 protein expression, respectively. Representative Western blots are shown. Parallel gels stained with Coomassie Blue G250 for equal protein loading evaluation were performed.

The p53 tumor suppressor protein is a key regulator of cell cycle arrest and of apoptosis. In response to DNA damage, the p53 is normally activated and accumulated to exert its DNA-binding activity for the regulation of genes involved either in G1 cell cycle arrest or apoptosis. To understand the mechanism behind the cell cycle progression in the presence of a genotoxic effect of Cd, we analyzed the p53 expression. At molecular level, we focused on the genes related to the p53 signalling pathway. Interestingly, the map of KEGG shows that the p53 gene was not regulated at the transcriptional level (Figure 2). At protein level, immunochemical results confirmed that there is no clear evidence of an upregulation of this transcription factor in Cd-exposed (0.1–10 *μ*M) samples (Figure 3).

**Figure 4:**
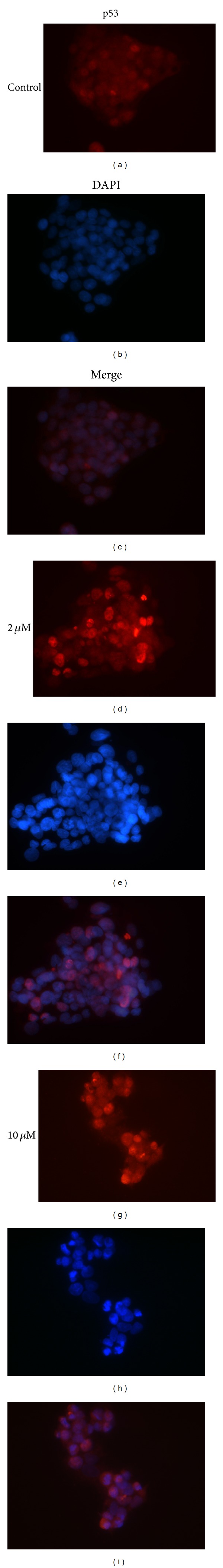
Visualization of p53 localization in CdCl_2_-treated HepG2 cells. Fixed and p53 stained cells were visualized by indirect probing with Alexa Fluor antibody. In control HepG2 (a), a spread distribution of the p53 is shown throughout the cells, while the treatment with Cd increases the nuclear localization of the protein (d, g). (a, d, g) cells probed for p53 in controls (a), 2 *μ*M (d), and 10 *μ*M (g) Cd concentrations. (b, e, h) cells stained with DAPI for the nuclei visualization in controls (b), 2 *μ*M (e), and 10 *μ*M (h) Cd concentrations. Merge is represented in (c, f, i) images. Microscopy magnification: 400x.

**Figure 5:**
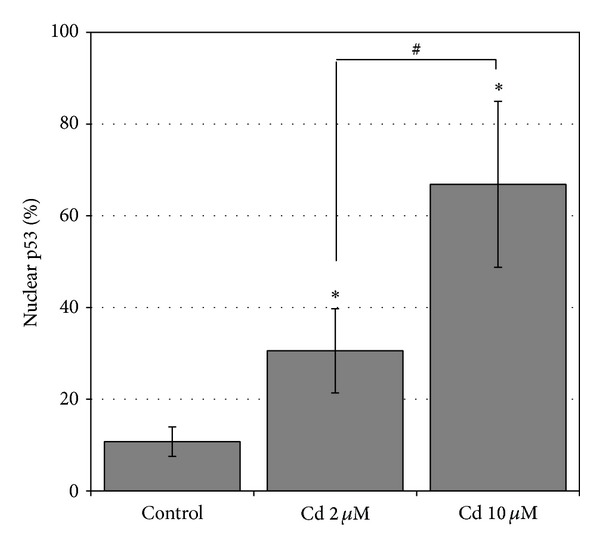
Quantification of the p53 nuclear localization. Percentage (mean ± SD) of HepG2 cells showing the p53 nuclear localization in the controls and in Cd-treated cells (2 and 10 *μ*M). The mean ± SD values of at least 3 independent samples are shown; a number of 400 < *n* < 1000 cells were counted. Significantly different from control: *P < 0.5; significantly different between Cd treatments: ^#^ *P* < 0.05.

We next analyzed the subcellular localization of p53 by indirect immunofluorescence in the presence of genotoxic Cd concentrations (2 and 10 *μ*M) to verify whether the p53 was correctly localized to exert its function. Fluorescence images showed that in control cells the p53 signal is spread and localizes throughout the cytoplasm (Figures 4(a)–4(c)). In cells treated with DNA-damaging concentrations of Cd (2 and 10 *μ*M), the p53 fluorescence signal is increasingly concentrated and accumulated in areas that co-localize with the nuclei (Figures 4(d)–4(f) and 4(g)–4(i)), as expected by a transcription factor. To quantitatively express the nuclear localization of the p53, a wide population (400–1000 cells/controls or Cd) of stained cells coming from independent experiments and different coverslips was counted. In controls only 10 ± 3,2% of the cells showed a p53 nuclear localization, confirming the predominant cytoplasmic distribution in unstressed cells, while in Cd-treated samples an increasing percentage of the cells showed that the transcription factor moves into the nucleus (Cd 2 *μ*M 30 ± 9,2%, and Cd 10 *μ*M 67 ± 18%) to activate downstream genes. The extent of p53 nuclear localization in controls and in Cd-treated cells is summarized quantitatively in Figure 5.

### 3.3. p21^Cip1/WAF-1^ Levels in Cd-Stressed HepG2 Cells

To better understand the p53 pathway and regulatory activity, we have analyzed the expression of the p21^Cip1/WAF-1^ protein. The p21^Cip1/WAF-1^ is a downstream protein known to be regulated also by p53 to trigger cell cycle arrest in DNA damaged cells. At gene level,* p21* ^*Cip1/WAF-1*^ was upregulated, as shown by the p53 signalling pathway (Figure 2, in red). Nevertheless, levels of p21^Cip1/WAF-1^ protein were comparable to controls at the lower Cd concentrations and decreased at 5 and 10 *μ*M Cd (Figure 3).

### 3.4. miRNA Modulation

We have analyzed the modulation of these small and noncoding molecules, acting at posttranscriptional level, to unravel the apparent contrast between the* p21* gene upregulation and the p21 protein downregulation at high Cd concentrations (5 and 10 *μ*M).

The effects of 10 *μ*M Cd on miRNAs were evaluated with microfluidic cards and the focus was on the upregulated miRNAs. In Cd-treated samples we identified two miRNAs that were upregulated: hsa-mir-138 and hsa-mir-372.

## 4. Discussion

The aim of this study was to investigate the role of the p53 tumor suppressor at gene and protein level in order to contribute to the comprehension of the reported resistance to apoptosis in Cd-treated cells [5]. Cadmium carcinogenicity has been recognized by epidemiological studies and animal experiments, as recently reviewed [6, 8]; however, the overall mechanism is still unclear. In addition, conflicting results concerning the effects of Cd on biological systems are present in the literature [13, 14], possibly justified by a recently demonstrated hormetic effect of this metal [24, 25]. Current evidence suggests that Cd carcinogenicity is not due to a direct genotoxic effect of this metal. Multiple indirect mechanisms, the interference with the cell response to DNA damage, the deregulation of cell growth, and the resistance to apoptosis are more accredited mechanisms which account for Cd carcinogenicity [5, 7, 11].

In HepG2 cells and, more in general, in mammalian cells cadmium exerts the genotoxic effect through reactive oxygen species generation, causing DNA strand breaks and chromosomal aberrations ([22], for a review see [5]). The DNA damage response is rigorously coordinated by multiple mechanisms, among which the p53 has a key role. The p53 is essential in the regulation of the cell cycle arrest and apoptosis for the processing of DNA damage and for restoring genomic stability or eliminating heavily damaged cells [26]. Our experiments demonstrated that in Cd-treated HepG2 cells, these regulatory mechanisms were not activated. Namely, no cell cycle arrest was induced at the highest Cd concentration tested (10 *μ*M), and no p53 upregulation was observed, as visualized at gene as well as at protein level. In response to stress, the p53 is normally accumulated in the nucleus and converted into an active DNA-binding form to control several sets of genes to prevent the proliferation of cells carrying a DNA damage [27]. To the best of our knowledge, our results of fluorescence microscopy show for the first time that the p53 was increasingly accumulated into the nucleus in Cd-treated HepG2 cells, according to the activity of a transcription factor. However, despite this correct localization in stressed cells, the p53 was not able to activate the downstream signals of cell cycle regulation and arrest to allow the DNA repair. This data is supported by the result on the p21^Cip1/WAF-1^, a p53 downstream protein responsible for the cell cycle arrest [26]. Indeed, in our samples the p21^Cip1/WAF-1^ showed levels comparable to controls or downregulated in Cd-treated HepG2. Therefore, this lack of regulation could account for the normal progression of the HepG2 cells into the cell cycle phases that we observed.

The uncontrolled proliferation of DNA-damaged cells and the acquisition of apoptotic resistance are important steps in the malignant transformation process. In this context, our results on the impairment of p53 pathway activity go through this direction. HepG2 cells, albeit derived from a human hepatoma, express a wild type and an inducible p53 activity, well documented previously [28]. However, previous works [3, 14] suggest that this activity could be impaired by conformational changes in the wild type p53 protein, as demonstrated in MCF7 and A549 cells exposed to soluble and particulate Cd compounds. The impairment of p53 activity could also be explained by our recent findings on microRNA (miRNA) levels in Cd-exposed HepG2 [29]. The miRNAs are small and noncoding RNAs which play a key role in gene expression at the posttranscriptional level, targeting mRNAs for cleavage or translational repression. We previously found that a large percentage of downregulated miRNAs belong to the let-7 family. The let-7 family is reported to have oncosuppressor functions as it regulates processes including cell division and DNA repair. In this regard, very interestingly it was recently described [30] that let-7a and let-7b expression are dependent on the p53 activity. Moreover, miRNAs are reported to have control activities on p21 at posttranscriptional level [31, 32] further sustaining our data on p21 expression at gene and protein level. Indeed, we suggest that the apparent contrast between* p21* ^*Cip1/WAF-1*^ upregulation at gene level and no modulation or downregulation at protein level could be possibly explained by the posttranscriptional activity of miRNAs. These findings support our data on the impaired function of this transcription factor. In order to further explain the lack of regulation of the p53 we analyzed the miRNA expression in Cd-treated HepG2. We analysed a panel of miRNA regulated upon stimulation of Cd, and we identified that two were upregulated after Cd exposure: mir-372 and mir-138, both connected to carcinogenesis [33, 34]. Wu and colleagues [32] showed that mir-372 can bind to the 3'UTR of p21^Cip1/WAF-1^ affecting its expression. Furthermore, the expression of mir-372 can promote cell proliferation and cell-cycle progression [34]. This mechanism warrants further investigation, but the work by Wu and colleagues together with our data on mir-372 upregulation provides hints on the involvement of miRNAs in the toxic mechanism induced by cadmium promoting proliferation activities. The second miRNA that we found upregulated, mir-138, was recently described to have oncosuppressor functions and its downregulation was associated with head and neck squamous cell carcinoma (HNSCC). However, while the deregulation of miR-138 is frequently observed in HNSCC and other cancer types, the exact role of miR-138 in tumorigenesis remains elusive [33]. Thus further functions and regulatory activities of this miRNA need to be deeply depicted.

Another possible mechanism that could contribute to the impairment of p53 activity and subsequent inhibition of the cell cycle arrest in HepG2 cells is the interference of metallothioneins (MTs). The expression and induction of this low molecular weight family of proteins are associated with protection against oxidative stress, cytotoxicity, and DNA damage. Cd is a transcriptional modulator, among others, of MT gene expression, and this family of metal-binding proteins appears also to play a key role in the prevention of apoptosis, acting as regulators of p53 folding and activity [35]. In addition, MT null cells are more susceptible to apoptotic death after exposure to apoptosis-inducing agents, and the involvement of MT in controlling apoptotic mechanisms was suggested in the past [36]. MTs were also proposed as proteins involved in the regulation of p53 stability and DNA-binding activity, along with a protective function in Cd-induced toxicity [37]. In HepG2 cells, MTs are strongly upregulated in the presence of Cd, as previously demonstrated by our group. [16, 21, 38]. These data sustain the speculation of a role for MT as negative regulators of apoptosis and of p53 activity [35].

## 5. Conclusions

**Figure 6:**
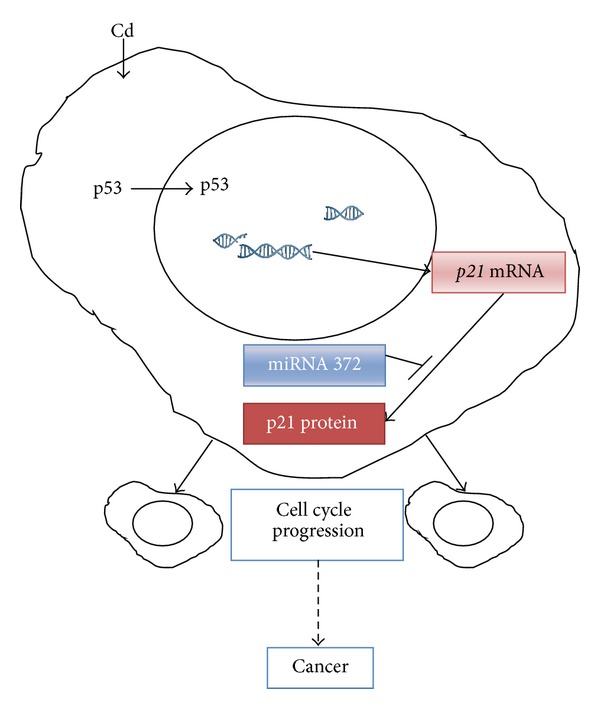
Overview of cadmium effects. (1) Cadmium is accumulated into HepG2 cells [21] and indirectly causes a genotoxic damage [22, 23] (data not shown); (2) the p53 is visualized into the nucleus, as expected, to exert its function of transcription factor; (3) the* p21* ^*Cip1/WAF-1*^ gene (*p21* mRNA) is upregulated, although no protein upregulation is observed possibly due to a posttranscriptional regulation by miRNA-372; (4) no cell cycle arrest is observed, thus leading to the transmission of DNA damage and ultimately to cancer.

In conclusion, as depicted and summarized in Figure 6, we demonstrated for the first time that in HepG2 cells exposed to Cd, the p53 was correctly moved and accumulated into the nucleus to exert its function of transcription factor. However, besides this correct nuclear localization, the signals for the cell cycle arrest were not activated. In this context, the p21^Cip1/WAF-1^, a p53 downstream protein and an important mediator of cell cycle arrest, was upregulated at gene level but not at protein level. These results could be explained by a posttranscriptional activity by the miRNA, as demonstrated by the upregulation of mir-372 in Cd-treated HepG2 cells, able to affect p21^Cip1/WAF-1^ expression and to promote cell proliferation. In this complex network, it seems of crucial importance to further investigate the relation between miRNA activity and p53 impairment.

The influence of Cd on p53 inactivation by conformational changes is under investigation by our group to more deeply elucidate the apoptotic resistance and the mechanism of Cd-carcinogenicity. Our results support the hypothesis of Cd as a double-edge sward factor [11], as it induces DNA damage and inhibits its repair.

## Abbreviations

CdCl_2_·H_2_O: Cd
FBS: Fetal bovine serum
MT: Metallothioneins
PMSF: Phenylmethylsulfonyl fluoride
SDS: Sodium dodecyl sulphate.
